# Simplivariate Models: Uncovering the Underlying Biology in Functional Genomics Data

**DOI:** 10.1371/journal.pone.0020747

**Published:** 2011-06-16

**Authors:** Edoardo Saccenti, Johan A. Westerhuis, Age K. Smilde, Mariët J. van der Werf, Jos A. Hageman, Margriet M. W. B. Hendriks

**Affiliations:** 1 Biosystems Data Analysis, Swammerdam Institute for Life Sciences, University of Amsterdam, Amsterdam, The Netherlands; 2 Netherlands Bioinformatics Centre, Nijmegen, The Netherlands; 3 TNO Quality of Life, Zeist, The Netherlands; 4 Biometris—Applied Statistics, Wageningen, The Netherlands; 5 Laboratory of Metabolic and Endocrine Diseases, Wilhelmina Children's Hospital, Utrecht Medical Centre, Utrecht, The Netherlands; 6 Netherlands Metabolomic Centre, Leiden, The Netherlands; University of Minnesota, United States of America

## Abstract

One of the first steps in analyzing high-dimensional functional genomics data is an exploratory analysis of such data. Cluster Analysis and Principal Component Analysis are then usually the method of choice. Despite their versatility they also have a severe drawback: they do not always generate simple and interpretable solutions. On the basis of the observation that functional genomics data often contain both informative and non-informative variation, we propose a method that finds sets of variables containing informative variation. This informative variation is subsequently expressed in easily interpretable simplivariate components.

We present a new implementation of the recently introduced simplivariate models. In this implementation, the informative variation is described by multiplicative models that can adequately represent the relations between functional genomics data. Both a simulated and two real-life metabolomics data sets show good performance of the method.

## Introduction

Functional genomics aim to obtain a complete overview of the biological response as a function of a biological perturbation that can be induced by given experimental conditions. The biological response can be, for instance, the expression levels of genes or metabolite concentrations. Functional genomics experiments are generally characterized by the generation of high-dimensional data.

One of the challenges in analyzing functional genomics data is the extraction of relevant biological information from such high-dimensional data sets, and to present this information in a simple and concise way to enhance interpretation. Exploratory analysis is usually a first step in such an analysis; examples are hierarchical clustering [1] and dimension reduction via principal components analysis (PCA) [2].

Exploratory analysis is often seen as providing an unbiased view of the data. However, a price has to be paid in terms of interpretability. For this reason, methods have been proposed that mix a certain amount of *a priori knowledge* with exploratory tools to attain more interpretable solutions. Examples of such methods are ASCA [3] and ANOVA-PCA [4] where the experimental design underlying the generation of the samples in the data matrix is explicitly imposed on the analysis thereby enhancing the interpretability of the results. These two methods are examples of utilizing *hard a priori knowledge* but such knowledge is not always available.

Our experience of analyzing functional genomics data sets over the years is that such data - broadly speaking - usually contains three major sources of variation: i) informative variation, ii) non-informative variation and iii) technical variation. Informative variation is defined as subsets of variables that show consistent and homogeneous covariation and are thus considered to reflect biological phenomena. The non-informative part consists of variables that show random and/or not biologically relevant systematic variation. The technical variation consists, for example, in sampling and measurement error. Hence, we want to find subsets of variables that show informative variation and discard all other types of variation. To fullfill our goal we recently introduced the idea of simplivariate models [5]. These models describe the informative variation by postulating that a studied biological phenomenon is not represented by all measured metabolites but only by a few subsets of such compounds. These subsets can be regarded as simplivariate components, each one accounting for a particular underlying biological phenomenon. A crucial aspect of the method is the choice of the model describing the relations between the metabolites in a simplivariate component. In the very first formulation additive models were used in an ANOVA-type fashion and when applied to metabolomics data they showed to be very effective in creating clusters of variables representing distinct biochemical processes. Because of the fact that an additive simplivariate component represents only metabolites belonging to the same process having mutual positive correlations, they do not have the full potential to model positively and negatively correlated metabolites. Indeed, correlations in functional genomics data reflect information on the relations in fold changes in metabolites, protein concentrations or expression levels. Hence, subsets of tightly correlated metabolites may hint to modules and regulatory motifs in the data.

To focus on modeling correlations, we implemented multiplicative simplivariate components as an example simple structure. Multiplicative models are also the basis of PCA, hence, this implementation is related to PCA. Several other extensions of PCA with 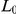, 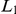 or 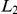 norm penalties on the loadings have appeared to reduce the number of variables in a principal component [6]. However, simplivariate models provide a flexible framework in which data can be analyzed according to a specific mathematical model chosen according to the problem being studied and in which the choice of the simplivariate components is data driven.

The method also gives a measure of the significance of a given simplivariate component by comparing it to a cluster of the same size which is randomly generated and in which the correlation structure arises purely by chance. This procedure is implemented to avoid overfitting due to chance correlations which is highly relevant in analyzing high-dimensional functional genomics data.

The remainder of the paper is structured as follows. General definitions and properties of simplivariate models are first presented together with examples of existing models and previous implementations which are discussed. The modeling of multiplicative structures is then introduced in a Singular Value Decomposition framework. The algorithm is illustrated in detail and general principles of Genetic Algorithms programming are introduced. The objective function for the proposed problem is illustrated together with the underlying necessary mathematical machinery. Finally, the performance of the methods is illustrated by means of simulations and two real-life NMR and GC-MS metabolomics data sets.

## Materials and Methods

### Simplivariate models

Simplivariate models have been first introduced in [5] and will be recapitulated in the following paragraph for convenience of the reader. Although the simplivariate framework was developed to aid the analysis of metabolomics data, it can be applied to any kind of platform as long as the variation in the measurements can be plausibly split into informative and non-informative variation. The traditional approach of breaking down variation in systematic variation and noise can be indeed too simple (or not hold at all) to analyze complex *omics* data. Simplivariate models are grounded on the observation that a data matrix **X** can be partitioned in components containing subsets of (biologically) related variables which describe experimentally measured entities such as metabolite concentrations, bucketed NMR spectra, expression levels of genes. This idea can be mathematically translated by considering that every element 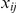 in **X** (where 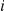 and 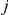 run over the rows and the columns, respectively) can be expressed as the sum of the contribution of different components:
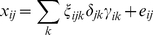
(1)where 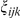 describes the informative parts of the data. In this context, the term 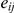 accounts for the non-informative part and should not be confounded with the residual random variation; 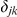 indicates the presence (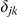 = 1, 0 otherwise) of the 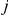-th variable in the 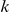-th simplivariate component and 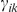 indicates the presence (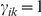, 0 otherwise) of the 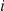-th objects in the 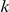-th simplivariate component. Equation (1) implicitly assumes that all the objects and/or variables in **X** can contribute to the 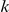-th simplivariate component but in this paper we will address only the case in which all objects contribute to all components (*i.e.* 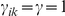) thus following a 1-way clustering approach. A remark on the utility of a 2-way clustering approach is given at the end of the Results and Discussion section. The formulation in Equation (1) allows, in principle, for overlapping clusters, in the sense that the same variable 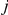 can appear in more than one simplivariate component. See Algorithm Implementation section for more details on overlapping components.

As 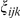 describes the relations between the objects and the variables in each of the simplivariate components, the actual form of 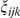 depends on the particular mathematical model chosen to model the data: the underlying idea is that biologically or functionally related variables can be modeled according to a specific mathematical model to be determined on the basis of the problem being studied. In this framework only subsets of variables contribute to those components, thus providing a final model which is of more simple interpretation, *i.e.* a simplivariate model.

Different mathematical models are available and some of them are routinely used in many statistical tools.

The most simple model is the *constant* model
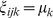
(2)where every simplivariate component 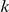 is equal to a constant 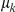. It is analogue to a two-mode clustering [7].

An *additive* model is given by
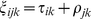
(3)and it is analogue to a two-way ANOVA decomposition of **X** [7]. This approach can be useful, for instance, when rows correspond to different experiments according to a given experimental design.

A *multiplicative* model
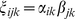
(4)is equivalent to a rank-1 component PCA decomposition of a selected subset of **X** and it will be the subject of this paper. This is the case when rows describe different individuals without a design. Combination of different kinds of models are also possible to form mixed models.

Many existing algorithms can produce simplivariate models according to the definition in Equation (1). In our first paper [5] we presented the implementation of both additive and multiplicative models in a simplivariate framework using two existing and well known algorithms. The additive model (3) was implemented in a plaid algorithm [8]–[10] which is a two mode clustering which looks for (possibly) overlapping clusters by iteratively searching the data to find patches of data that can be modeled by means of an ANOVA decomposition. The multiplicative model (4) was implemented using interpretable dimension reduction (IDR) [11] which is an algorithm that starts from the standard PCA solutions and, by reducing and summarizing the number of non-zero elements of the loading vector, produces a new sparse loading vector which is simpler to interpret.

**Figure 1:**
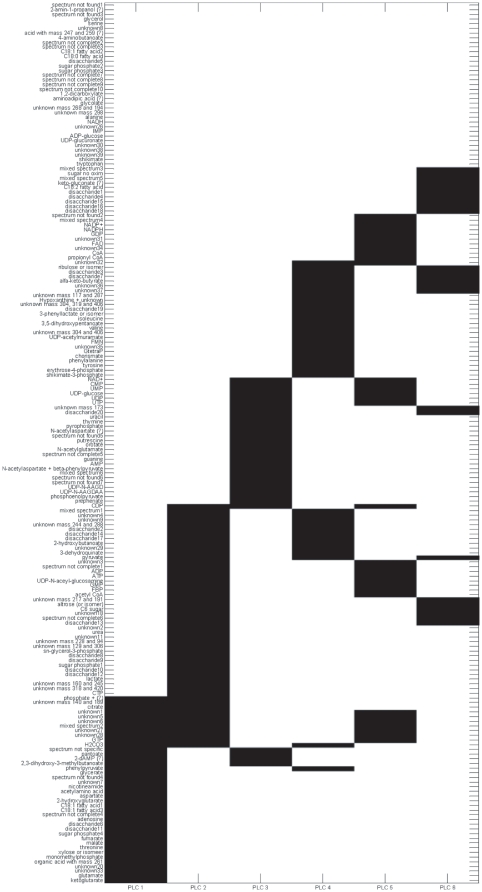
Plaid decomposition of the *E. coli* data set (see section GC-MS metabolomics data set for a description) implementing aa additive simplivariate model as in **Equation 3**. Figure reproduced from [5].

**Figure 2:**
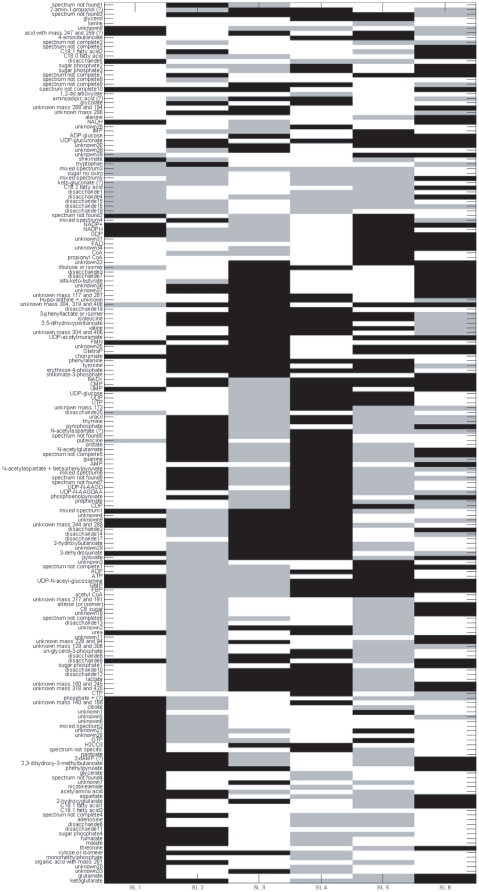
IDR decomposition of the *E. coli* data set (see section GC-MS metabolomics data set for a description) implementing a multiplicative simplivariate model as in **Equation 4**. Figure reproduced from [5].

Plaid was shown to be effective in producing clusters with distinct biochemical meaning while IDR resulted in clusters containing too many metabolites to be of any practical utility: the resulting simplivariate components were not *simple* enough to provide a straightforward biological interpretation. These results are reproduced in Figure 1 and 2, showing the plaid decomposition (additive model) and the IDR decomposition (multiplicative model) of the *Escherichia coli NST 74* GC-MS data set which will be also used in this paper. For a discussion of the biological interpretation see [5].

Unfortunately plaid also has several drawbacks, the main being that ANOVA-type simplivariate components do not have the potential to model negative correlations so that an important part of the relationships among variables is missed. Additive models can only describe similar sized variations in different entities (such as metabolites or enzymes) while multiplicative models can account for correlation structures in the data. Correlations in *omics* data are important as they can reflect information on fold changes in metabolite, protein concentrations or expression levels and to describe individual cases of mutual regulation by metabolites/genes that can result in the definition of metabolic or gene regulatory networks. For this reason, the introduction of a new algorithm, able to fully model correlations like IDR while retaining the clarity of results, was deemed necessary.

### Modeling multiplicative structures

According to the philosophy of simplivariate models, we aim to partition a given data matrix **X**, of size *J* variables and *I* objects, into a (large) non-informative part and in 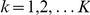 informative partitions 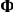 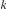 (whose elements are 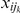) that can be modeled with a predefined mathematical model able to take into account the correlation among the variables. 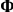 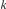 are then subsets of the columns of **X**.

Given a data matrix **X**, correlations among variables (columns) can arise when they describe, for instance, metabolites belonging to the same metabolic pathway or network or related physico-chemical entities like peaks of the same molecule in an NMR spectrum. These correlations translate into sets of correlated variables, each set representing some physical and/or chemical process. The assumption is that the correlation among this subset of variables is the outcome of one underlying latent phenomenon. This correlated set of variables can then be modeled with a simple multiplicative model.

The final goal is to obtain partitions 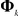 of **X** that can be modeled by means of multiplicative simplivariate components (SC):
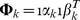
(5)where 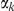 and 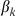 are vectors of size 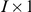 and 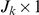 respectively.

According to the Singular Values Decomposition (SVD) theorem [12], any matrix **A** of size 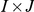 can be approximated with a rank-1 singular value decomposition as follow:
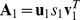
(6)where 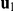 and 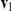 are the first singular vectors of size 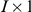 and 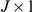 respectively, and 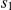 is the corresponding largest singular value. By exchanging a general matrix 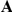 with the 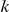-th subset 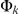 of **X** in Equation (6), it can be written
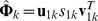
(7)where 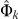 indicates the rank-1 SVD approximation of 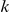-th subset 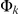 of **X**. Rearranging the singular vector multiplications by combining the singular value and the vector 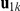 in such a way that
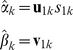
(8)Equation (7) becomes
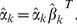
(9)By comparing Equations (9) and (5) it appears that a rank-1 singular value decomposition is a natural choice for modeling multiplicative structures. The search for subset 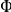 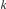 of size 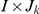 is translated into the search of groups of variables that can be fitted (*i.e.* approximated) by means of a rank-1 SVD. Incidentally, it should be noted that a rank-1 SVD also has the property of being optimal in the sense that a matrix is approximated with minimum least squares error [13].

### Algorithm Description

#### Search strategy

We are searching for subsets 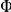 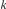 of size 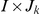 by estimating variable memberships of a simplivariate component. This can be achieved through the maximization of the sum of squares 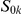 over all elements of 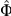 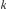. In other words this means looking for cluster of variables that can be best approximated by the multiplicative model, that is selecting the set of variables for which the rank-1 approximation makes sense. It holds
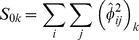
(10)where 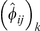 indicates the elements of the 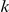-th fitted simplivariate component 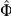 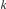.

Ideally, the maximization is over all possible subsets of variables of sizes in-between 2 and 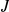 that can be formed from the 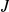 variables in **X**. Given 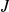 variables there are 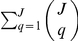 possible subsets. (See Text S1, Section S2). Due to its combinatorial nature, this is an NP-hard problem and the time needed for this task increases exponentially with the number of variables [14]. Genetic Algorithms (GA) can be a convenient approach to screen a large numbers of solutions [15].

#### Genetic Algorithm

Genetic Algorithms are a class of global optimizers and rely on the maximization of an objective function which may depend on several parameters. GA's search the parameter space to find an optimal solution, avoiding the risk of being trapped in a local minimum (maximum). In addition, finding the best subset of variables to construct 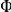 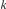 aiming for the largest 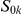 leads to a mixed binary optimization problem. This problem cannot be solved with standard methods like least squares but can be overcome by, amongst others, a GA approach; an integer type coding can indeed be written for this kind of algorithm [7].

Although many different implementations of GA's exist, several steps are equal for all GA's. We follow the schema given in [7] for a brief outline of a GA optimization procedure and refer the reader to [16] and [17] for an exhaustive review of principles and practice of Genetic Algorithms. A GA optimization procedure can be summarized as follow:

1. *Initiliazation*: The GA operates on groups of solutions at a time. A group of random solutions (population) is generated. These random solutions are vectors of class membership labels (where 1 indicates that a variable belongs to a given SC and 0 otherwise) randomly chosen from a collection of random vectors containing different percentages of 1 and 0 to assure maximum representativity.
2. *Evaluation*: The SC is evaluated by means of an objective function (OF). The objective function evaluates the quality of the solutions and expresses it with a single number. The OF is custom made and needs to be tailored according to the specific problem under study. This topic is specifically addressed below in the section Objective Function. Summarizing, the objective function evaluates how well the found simplivariate component 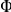 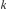 can be fitted to a rank-1 SVD as presented in Equation (9).
3. *Stop*: The GA usually stops when a maximum number of generations is used or when the improvement of the solution is below a predetermined threshold.
4. *Selection*: A given percentage of the best solutions in a population are selected to form the next generation.
5. *Recombination*: A new population is formed by combining two selected existing solutions (parents) to give birth to two new solutions (children).
6. *Mutation*: A part of a solution is randomly selected and mutated. For instance a 0 can be turned to 1 or *vice-versa*. The mutation rate is usually kept low to avoid random behavior.

#### Algorithm implementation

The overall algorithm can be summarized in the following way:

1. Autoscale the original data matrix **X**.
2. Find 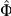 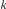 using the Genetic algorithm search.
3. Subtract 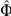 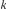 from the corresponding columns of **X**. If 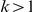 apply a backfitting procedure for each obtained component 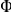 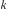 without changing the variable memberships.
4. Repeat steps 2. to 4. for 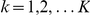.

Some comments on points 1, 3 and 4 of the previous algorithm outline.

1. Since the aim is to model correlations among variables, the matrix **X** is autoscaled [18], [19]. *Autoscaling* means that each column of the data matrix **X** is subtracted by its mean and divided by its standard deviation. This procedure is sometimes called standardization or *z*-scoring. Additionally, autoscaling assures that variables with smaller variance have the same *a priori* chance to be selected, without further adjustments of the objective function.

3. Backfitting is a well established procedure [18] and it is applied to improving the fit of the model. Each simplivariate component is fitted to the residual from the model excluding the simplivariate component selected. When the 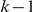-th component is found (with 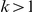) the 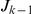 columns of 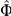 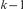 are subtracted from the corresponding columns of **X** in a such a way that
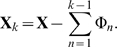
(11)The 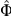 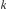 is re-estimated from 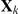 with a rank-1 SVD according to equations (6)–(10). The next simplivariate component is then searched on 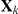 (that is **X** is set equal to 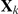 in the algorithm).

The backfitting can affect the fit of the chosen simplivariate components to the data in case of overlapping components. This procedure does not alter the set of variables that compose the components that have been selected in previous iterations. More precisely, the backfitting performed after obtaining the *k*-th component will not influence the subsets of variables in components 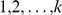, but may influence the choice of variables in a component for larger *k* when variables are shared between these components and components 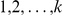.

4. Simplivariate models serve as an exploratory tool. Determining the exact number of significant clusters that can be inferred from a data set is out of the scope of the simplivariate methods and dedicated methods such as the Bayesian Information Criterion [20], GAP statistic [21] and the knee method [22] have been introduced for this purpose. Nevertheless, implementations of simplivariate models in algorithms aiming to detect the actual number of clusters in a data set can be possible. The choice of the final number *K* of components to retrieve is somehow arbitrary, although the algorithm offers a measure of the importance of the *k*-th simplivariate component. This aspect is discussed in the Objective Function section, particularly in the Subsections dedicated to the reference distribution 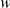 and to the Scaling Term *T*. A possible criterion to asses the ultimate value of 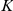 is introduced in Results and Discussion section dedicated to the discussion of a simulated data set. We did not investigated the ability of the method of assessing the real number of clusters in the data set and for convenience we presented results up to 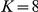 similarly to what was presented in [5].

This algorithm can in principle be applied to data sets of any size. As all objects (rows) of the data matrix contribute to a simplivariate component, the computational time depends solely on the number of variables in **X** and on the number 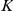 of simplivariate components one aims to retrieve.

The algorithm allows for overlapping components. This means that the same variable(s) can be found in one or more simplivariate components. Although this is an indication of the versatility of the method, overlapping components do not necessarily translate into more accurate, significant or informative results. As a matter of fact, overlapping components are not easy to (biologically) interpret. For instance, a PCA model consists only of overlapping clusters (*i.e.* every variable contributes to every principal component) and therefore is very difficult, if not impossible to interpret. The same problem arises when analyzing results from the IDR and Plaid algorithm as shown in Figures 1 and 2. In the Results and Discussion Section we show how simplivariate components are much more readable and easy to interpret than plaid or IDR solutions.

### Objective Function

#### Objective Function

The maximization of the sum of squares 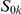 is a trade off between selecting simplivariate components based on a large number of variables which may give a high sum of squares and selecting smaller sets of more homogeneous variables that better fit the proposed model.

Three features complicate the optimization process. First, 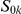 will almost always increase when adding an additional variable. Second, the combinatorial nature of the problem, paired with the properties of the distribution of 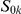 values, can bias the GA's solutions towards clusters of size 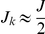. Third, high correlations can occur by chance, generating simplivariate components with a very small number of variables. Considering the number of variables that will form the simple components, these three phenomena bias the maximization of 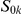 in different and counteracting ways. There are no easy cures of these problems. We tackled these problems by penalizing and scaling the objective function.

We devised an objective function 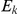 which consists of two terms
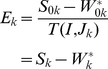
(12)where 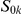 is the sum of squares of the elements of 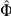 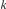, 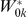 is the penalization term for chance correlations and 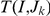 is the scaling term. The latter terms are of the utmost importance. The rationale behind their introduction and their role is explained in details in the next three sections. Summarizing, the (standardized) fit value (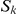) of the original data (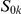) is confronted with the (standardized) distribution of fit values of random data components (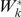) of the same size, an idea which is related to the gap statistic [21]. Hence, the distance (or gap) between 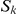 and 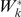 can be seen as a measure of significance of the *k*-th simplivariate component. In other words, the reference distribution 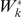 acts as an (empirical) null distribution to test the null hypothesis 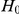 that 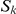 of a given simplivariate component 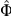 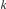 is equal to that of a cluster of the same size which is randomly generated and in which the correlation structure arises purely by chance.

#### Correction for chance correlations

The occurrence of chance correlations is well known: it increases when the number of observations is small compared to the number of variables (as almost usually happens in the case of *functional genomics* datasets) [23] and can become critical when building models for subsets of a larger set of variables [24]. Chance correlations therefore influence the maximization of 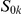. We compensate for this effect by introducing the correction term 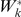 which is extracted from a reference distribution 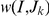. The correction term is such that smaller clusters are penalized more than larger ones, counteracting the bias of the simplivariate component estimation procedure towards smaller components as caused by chance correlations.

#### The reference distribution 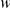


The reference distribution describes the variation in the sums of squares 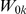 of simplivariate models of size 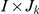 fitted to random data, accounting for complete absence of structure [253]. The number of variables 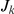 and objects 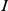 that are used to construct the distribution 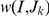 equals those that underly the simplivariate component that resulted in the specific value of 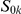 (which also equals the size of 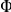 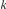). Since not only the location but also the scale of the distribution is related to the size of the simplivariate component (*i.e.* 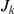), the penalty 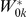 is estimated as the 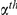 percentile of the distribution 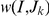. The choice relies on the fact that the percentile is not sensitive to extreme values of the distribution tails and can be easily numerically computed once the reference distribution has been generated by using the percentile definition [26]. This is actually similar to test the null hypothesis 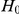 (*i.e.* the correlation structure of 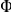 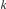 is due purely to chance correlations) with a 0.01 confidence threshold.

The reference distribution 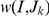 and 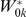 can be derived both empirically and theoretically. We choose to derive the distribution 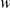 from randomly generated subsets of sizes 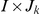 in the range 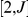 by permutations of the original data matrix **X**. This is equivalent to randomly generating sets of autoscaled variables. This choice is based on the need of reducing the computational burden required by the GA while exploiting at maximum the versatility and the power of the GA approach. More details are given in File S1. Results presented here have been obtained with the common 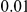 percentile but more conservative values can of course be used as long as a proper number of permutations is applied to sample the distributional tails [27].

#### The scaling term 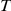


The scaling factor 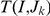 corrects for the combinatorial/probabilistic bias towards larger components. The rationale behind this correction can be expressed in terms of probability theory and results from random matrix theory. The mathematical and theoretical machinery is explained in File S1.

Table 1 contains a summary of mathematical the notation and symbols used through the paper.

**Table 1:** Summary of mathematical notation and symbols.

**X** (matrix)	bold uppercase
**x** (vector)	bold lowercase
*x* (scalar)	italic
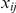	element 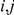 of a matrix **X**
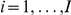	object index
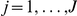	variable index
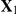	Rank-one singular value decomposition (SVD) of a matrix **X**
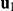	Rank-one singular vector of size 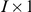
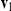	Rank-one singular vector of size 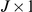
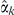	Singular vectors re-arrangement: 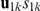
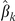	Singular vectros re-arrangement: 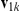
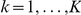	simplivariate component index
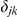	simplivariate component 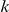 class membership for variables
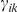	simplivariate component 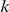 class membership for objects
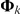	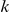 -th cluster of size 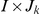 formed by 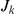 columns of **X**
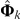	Rank-1 Singular Value Decomposition of 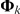
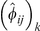	Elements 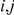 of 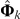
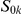	Sum of squares over the elements of 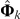
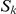	Standardized 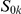
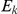	Objective function for the 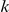 -th simplivariate component
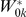	Penalization term for chance correlations
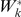	Standardized penalization term for chance correlations
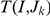	Scaling term
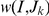	Reference distribution for the variation of the sum of squares for random fitted data

### Software

The algorithm was programmed in Matlab 7.1 R14 [28] and the Genetic Algorithm and Direct Search [29] Toolbox was used for the Genetic Algorithm implementation. All GA runs were executed five-fold with different random seeds to exclude any (un)lucky starting positions. The results from the five runs should be similar and the best solution is chosen.

All calculations were performed on an AMD Athlon XP 2400+ 2.00 GHz 512 MB RAM PC running Windows XP.

The Matlab m-files of the method presented can be downloaded from www.bdagroup.nl.

## Results and Discussion

### Simulated dataset

The method will be first applied to a simulated dataset 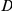 of size 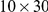 in which four multiplicative structures 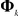, 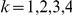 of size 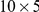 have been added to a background random noise matrix **B** of size 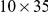. A heat map of the simulated dataset **D** is shown in Figure S1. Structures 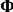 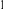, 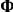 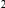, 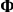 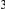 and 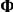 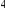 contain features 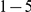, 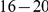, 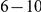 and 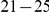 respectively, that can be intended to represent biological entities, *e.g.*, groups of biological related metabolites.

These structures are in the form 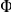 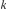 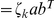 where 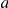 and 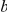 are random vectors drawn from a standard normal distribution and 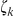 is a positive real number.
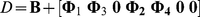
(13)where **0** is a zero matrix of size 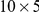.

**Figure 3:**
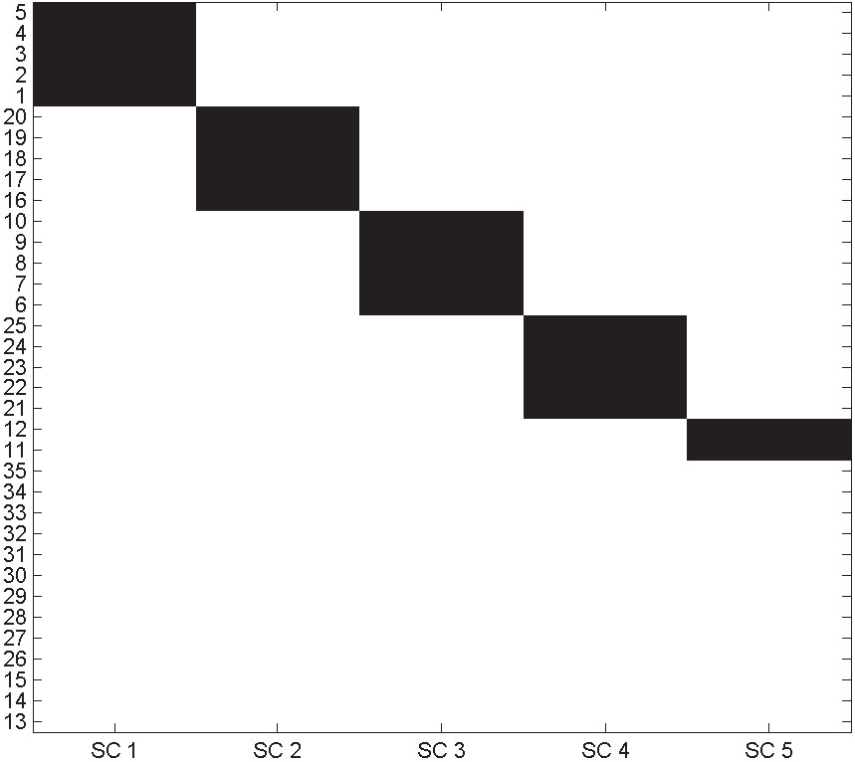
Dataset decomposition obtained by means of a multiplicative model implemented in the algorithm described in the Methods section. Black squares indicate that a certain variable belongs to a given simplivariate component (SC). The algorithm is able to retrieve four simplivariate components (referred as SC 1, 2, 3, 4, 5) containing sets of correlated variables.

Each structure 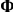 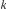 is purely multiplicative and can be modeled by 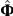 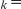 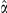 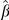 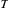, that can be decomposed in one loading and one score vector by means of a rank-one singular value decomposition as described in the Methods section (Equation 6). The proposed method is able to recover the four structures containing correlated variables as shown in Figure 3. A summary of the statistics is given in Table 2 The order in which the four structures are recovered [1]–[5], [16]–[20], [6]–[10], [21]–[25] reflects the strength of the correlation introduced in the simulated dataset: 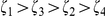. It is interesting to note how for the fifth simplivariate component, the value of 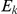 becomes negative, indicating the non significance of that component: this component is indeed formed by chance correlation of two background noise variables.

**Table 2:** Summary of statistics parameters for the decomposition of the a simulated data set.

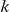	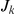	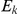	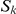	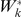
1	5	0.4294	1.0000	0.5706
2	5	0.4292	0.9999	0.5706
3	5	0.4289	0.9995	0.5706
4	5	0.4286	0.9992	0.5706
5	2	−0.2713	0.5856	0.8569

20 out 35 variables have been selected. The fifth simplivariate component is shown for completeness (see text).

### NMR metabolomics dataset

As a first example, we choose a data set which is part of the Metref data set [30], [31]. Forty urine samples from the same individual (male, 35 year old) have been collected over a period of two months and subjected to 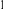
H Nuclear Magnetic Resonance spectroscopy profiling on a 600 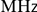 spectrometer. Details about samples collection, preparation, NMR experiments and spectra precessing can be found in [31]. Processed spectra have been subjected to 0.02 ppm bucketing, and obtained data has been summarized into a 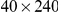 points data matrix.

An NMR spectrum of a urine sample or other biofluid can be regarded as the superposition of the spectra of tens to thousand small molecules of low or very low molecular weight. This reflects the well known complex correlation structure of NMR data sets: correlations among resonances from the same molecule appear together with correlations occurring among peaks of different molecules that covary because they occur in the same biological process (e.g. the same metabolic pathway).

Ideally, the correlation between resonances from different molecules would be high but usually not as strong as resonances from the same molecule. However, background noise and overlap of non-related signals, may result in the lowering of correlation strengths and in the appearance of spurious correlations between peaks [32]. Analysis of NMR data by means of the analysis of correlations is therefore a challenging task; the heat map of the correlation structure of a pool **X** of 40 human urine NMR spectra is shown in Figure S2.

**Figure 4:**
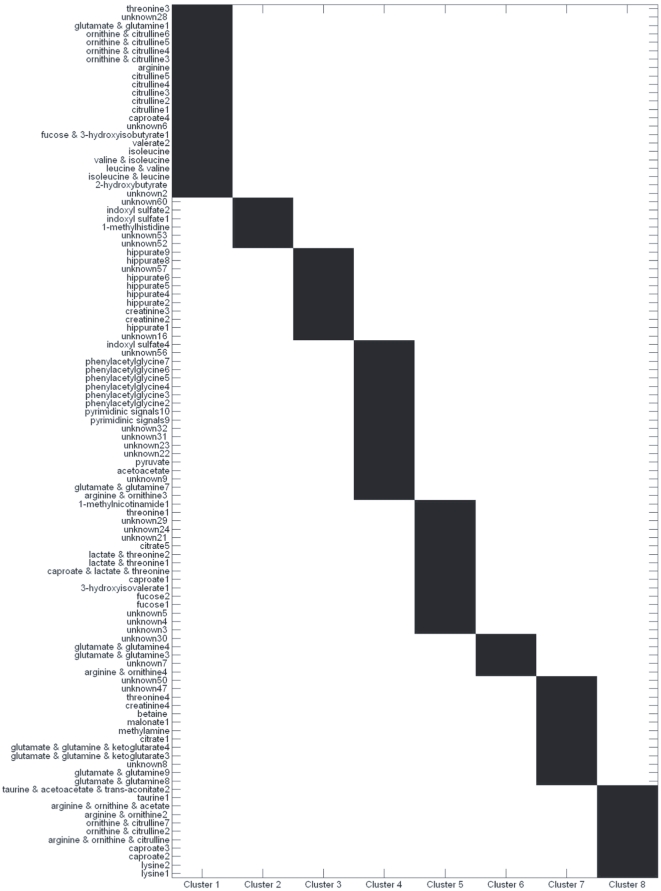
First eight simplivariate components from the multiplicative simplivariate decomposition of the NMR human urine multiple collection data set. Results are grouped as much as possible for clarity and non selected metabolites are not shown. See test for details on the biological interpretation.

When applied to the Metref NMR dataset, our methods performed well, generating simplivariate components with a distinct biochemical and biological meaning. Summary statistics for the first eight simplivariate components (SC) is given in Table 3 while Figure 4 gives a graphical illustration of the metabolite composition of the SC's. In general, each SC contains resonances arising from molecules in the same metabolic pathway as well as of resonances from the same molecule. It is interesting to note (see Table 3) that the value for the sum of squares 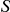 is not decreasing. SC 2 has a larger 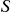 value than SC 1 but has a smaller size (6 variables 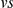 23): it is much more penalized, resulting in a lower 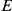 value.

**Table 3:** Summary of statistics parameters for the decomposition of the NMR metabolomics data set.

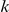	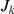	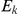	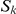	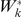
1	23	0.6582	0.8050	0.1468
2	6	0.6399	0.9793	0.3394
3	11	0.5885	0.8169	0.2285
4	19	0.5737	0.7376	0.1639
5	16	0.5669	0.7483	0.1814
6	5	0.5169	0.9012	0.3843
7	13	0.5104	0.7162	0.2059
8	11	0.5057	0.7342	0.2285

114 out 240 variables have been selected.

Without going into all the details it is interesting to see what kind of information can be extracted from the simple components. As an example, SC 1 contains resonances of different essential and non-essential aminoacids like arginine, citrulline, glutamate, glutamine, isoleucine, leucine, ornithine, threonine together with peaks of short chain fatty acids like 2- and 3- hydroxybutyrate. Citrulline, ornithine and arginine are subproducts of the urea cycle [33].

Analysis of SC 3 shows how our method is able to model also negatively correlated metabolites. SC 3 contains peaks from hippurate and creatinine (plus two unassigned resonances), two urinary metabolites whose clearance is known to be negatively correlated in healthy subjects [34] (See also Figure S3).

Simplivariate component 4 contains signals from phenylacetylglycine (PAG) and indoxyl sulfate (IF), two metabolites related to the activity of gut microbiota: PAG has only recently been attributed to gut microflora [35], while IF is a uremic toxin produced in the liver from indole, which is a subproduct of tryptophan bacterial metabolism [36], [37]. In addition, both pyruvate and indole are involved in tryptophan degration through an 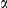,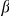-elimination reaction [38]. Further, acetoaceate is also a subproduct, together with pyruvate, of tryptophan catabolism [39]. Both pyruvate and acetoacetate are intermediates of glycolysis [33].

In SC 5 we found again resonances form energy associated metabolites [40] like 1-methyldicotinamide and lactate (which overlaps with the threonine resonances) and peaks from fucose. Interestingly, glycopeptides containing fucose and threonine have been observed in human urine [41], [42].

### GC-MS metabolomics dataset

*Escherichia coli* NST 74, a phenylalanine overproducing strain and *E. coli* W3110, a wild type strain, were grown in batch fermentations at 30

C in a Bioflow II (New Brunswick Scientific) bioreactor as previously described [43]. Cells were cultivated on MMT12 medium with glucose as carbon source, a constant pH and a constant oxygen tension of 30%. Samples were taken at 16, 24, 40 and 48 hours and analyzed by GC-MS and LC-MS. Peaks related to the substrates used for growth (glucose and succinate) were removed from the data. The resulting data set consisted of 28 measurements and 188 metabolites. Extensive details on experimental setup, GC-MS and LC-MS analysis and subsequent preprocessing can be found in [43].

**Figure 5:**
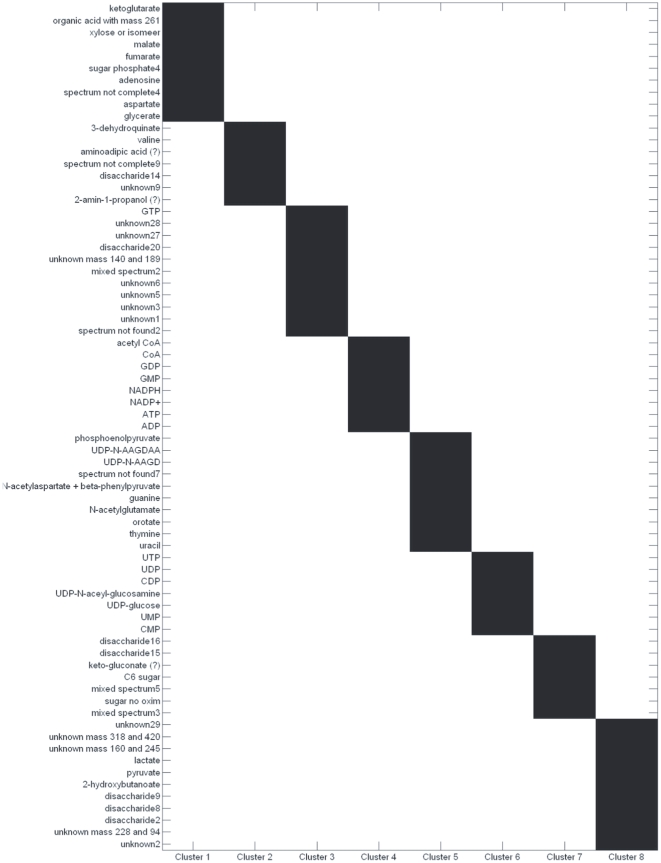
First eight simplivariate components from the multiplicative simplivariate decomposition of the GC-MS *E. coli* data set. Results are grouped as much as possible for clarity and non selected metabolites are not shown. See text for details on the biological interpretation.

When applied to this dataset, the method is able to retrieve biologically correlated metabolites in small sized simplivariate components. Results are graphically displayed in Figure 5 while a statistics summary is given in Table 4. Metabolites belonging to the Krebs' cycle (2-ketoglutarate, fumarate and malate) are found in SC 1, similarly to what was found in [5].

**Table 4:** Summary of statistics parameters for the decomposition of the GC-MS metabolomic dat set.

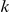	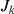	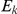	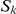	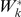
1	10	0.5983	0.8581	0.2599
2	7	0.5440	0.8646	0.3206
3	11	0.5042	0.7504	0.2426
4	8	0.4666	0.7626	0.2959
5	10	0.4510	0.7109	0.2599
6	7	0.4349	0.7555	0.3206
7	7	0.4196	0.7402	0.3206
8	11	0.4084	0.6545	0.2462

71 out 188 variables have been selected.

Simplivariate component 4 contains molecules that are fundamental participants in many metabolic reactions such as carbohydrate metabolism or fat metabolism.

Three metabolites (N-acetylglutamate, N-acetylaspartate and 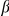-phenylpyruvate) that have been demonstrated to specifically correlate with the phenylalanine production titer [44] are found in SC 5. Simplivariate component 5 also contains UDP-N-AAGD and UDP-N-AAGDAA, cell wall precursors for peptoglycans biosynthesis [45] together with uracil, thymine and guanine, the three nucleobases whose concentration is above the detection limit in this data set.

Nucleotides (CMP, UDP, UTP, CDP, UMP, UDP-glucose, UDP-N-Acetyl-glucosamine) involved in cell wall biosynthesis and in the cell wall machinery [46] are clustered together in cluster 6. Metabolites related to lactate fermentation such as pyruvate and lactate are found in SC 8.

This survey of the retrieved SC's allows us to point out a subtle point which is too often neglected when analyzing a dataset on the base of correlations. We expected to retrieve the complete phenylalanine biosynthesis pathway (erythrose-4-phosphate, 3-dehydroquinate, shikimate-3-phosphate, chorismate, phenylpyruvate, and phenylalanine itself) and several compounds which are side routes of this pathway, (*i.e.* 3-phenyllactate and tyrosine), but we could only get a tight SC containing chorismate, phenylalanine and tyrosine (SC 10, not shown). We found out that these metabolites show low/moderate correlations: actually only the concentrations of chorismate and phenylalanine show a moderately strong correlation (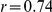). Phenylpyruvate shows correlation (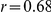) with chorismate, but it is found in SC 12 (not shown) together with 2-hydroxyglutarate with whom it has a stronger correlation (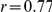). The concentrations of all others metabolites show low or no correlation at all.

This fact can be explained by considering the particular experimental design underlying the generation of this data set that contains different strains in different growth conditions. It must indeed be borne in mind that some metabolites, measured in different conditions, can be far from a steady state and this can result in the alteration of correlation patterns [47], hindering the interpretation of results in the case of metabolomics data [48]. Indeed, if one considers only samples 25–28 (NST 74 strain, oxygen 30%, pH 7.0, phosphate concentration 1; see [43] for details), a strong correlation between chorismate and prephenate concentrations (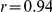) can be observed while those metabolites do not correlate in the complete dataset (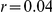). See Figure S4 for a heat map of the correlation structure of the phenylalanine pathway.

When applying an additive model [5], the phenylalanine pathway was retrieved at the cost of very large simplivariate components (on average larger than 40 metabolites). Our method has the advantage to produce tight clusters, accounting for more precise underlying biological effects, which are more easily interpretable.

It is clear that with respect to a particular experimental design, some metabolic pathways can be modeled with a simple multiplicative model only if the sampling design is taken into account. This can be done by extending this method to 2-way data clustering, by searching the best combinations of variables and samples that maximize the objective function. These extensions will be the subject of a follow-up paper.

### Overall remarks

As remarked in the Material and Methods section, the proposed method is closely related to Principal Component Analysis and IDR. Figure 2 shows the IDR implementation of the multiplicative model (see [5] for PCA results, in particular Figure 4). It shows that all components have contributions from all metabolites. This fact impairs a straightforward biological interpretation of the results and indicates at the greatest extent the need of simplicity that can be attained in a simplivariate framework. As a conclusive remark we can note that we did not obtain overlapping clusters although no restrictions on this aspect are imposed neither by the multiplicative model chosen to fit the data or by the particular implementation (GA based) of the algorithm. This is likely due to the larger number of variables in respect to the small number of clusters.

### Conclusions

Simplivariate models are presented as a new framework for exploring high-dimensional functional genomics data constrained by *soft a priori knowledge* to arrive at meaningful solutions. Any user-defined simple structure can be imposed and in this paper a simple multiplicative structure was chosen. The simulations show that the method does what it is supposed to do. The algorithm is based on natural computation thereby avoiding problems of local minima. Moreover, the optimization criterion used to fit the model explicitly selects significant components. The method is illustrated with an NMR and an MS based metabolomics data set. In both cases, the methods produce interpretable simplivariate components. The method can be used for analyzing any functional genomics data set where the underlying assumption of partitioning of informative and non-informative variation holds.

## Supporting Information

Figure S1
**Heat map of a simulated dataset** ***D*** **containing four correlated structures (variables** 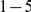 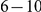 **,** 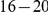 **and** 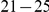 **).**
(EPS)

Figure S2
**Heat map of the correlation structure of a pool** ***X*** **of 40 human urine NMR spectra.** The statistical correlation matrix 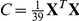 shows the highly correlated nature of NMR spectra.(EPS)

Figure S3 **Correlation pattern within hippurate peaks is shown, together with the anti-correlation between creatinine and hippurate.**
(EPS)

Figure S4 **Heat map of the expected correlation pattern for the phenylalanine biosynthesis pathway for the NST 74, a phenylalanine overproducing strain and for the wild type strain.**
(EPS)

File S1
**Detailed explanation of the mathematical and theoretical machinery underlying the reference distribution** 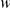 **and the scaling term** 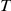 **.**
(TEX)
